# The Pre-Treatment C-Reactive Protein Represents a Prognostic Factor in Patients with Oral and Oropharyngeal Cancer Treated with Radiotherapy

**DOI:** 10.3390/cancers12030626

**Published:** 2020-03-08

**Authors:** Olivia Knittelfelder, Daniela Delago, Gabriele Jakse, Katarzyna Lukasiak, Eva-Maria Thurner, Dietmar Thurnher, Martin Pichler, Wilfried Renner, Heidi Stranzl-Lawatsch, Tanja Langsenlehner

**Affiliations:** 1 Department of Therapeutic Radiology and Oncology, Comprehensive Cancer Center, Medical University of Graz, 8036 Graz, Austria; o.knittelfelder@hotmail.com (O.K.); daniela.delago@stud.medunigraz.at (D.D.); gabriele.jakse@klinikum-graz.at (G.J.); katarzyna.lukasiak@klinikum-graz.at (K.L.); Eva-Maria.Thurner@klinikum-graz.at (E.-M.T.); heidi.stranzl@medunigraz.at (H.S.-L.); 2 Department of Otorhinolaryngology, Medical University of Graz, 8036 Graz, Austria; dietmar.thurnher@medunigraz.at; 3 Division of Oncology, Department of Internal Medicine, Medical University of Graz, 8036 Graz, Austria; martin.pichler@medunigraz.at; 4 Division of Cancer Medicine, Department of Experimental Therapeutics, The University of Texas MD Anderson Cancer Center, UTHealth, Texas A&M College of Medicine, Houston, TX 77030, USA; 5 Clinical Institute of Medical and Chemical Laboratory Diagnostics, Medical University of Graz, 8036 Graz, Austria; wilfried.renner@medunigraz.at

**Keywords:** oral and oropharyngeal cancer, biomarker, inflammation, C- reactive protein (CRP), prognostic factor, outcome

## Abstract

The purpose of the present study was to evaluate the prognostic significance of the pre- treatment C-reactive protein (CRP) level in a cohort of 503 patients with oral and oropharyngeal cancer treated at a tertiary academic center between 2000 and 2017. Cancer-specific survival (CSS), overall survival (OS) and loco-regional control (LC) were calculated using Kaplan-Meier analysis. To evaluate the prognostic value of the CRP level for the clinical endpoints, univariate and multivariate Cox regression models were applied. The median follow-up period was 61 months. Patients were divided into elevated CRP (≥5 mg/L) and normal CRP groups, according to pre-treatment plasma levels. An increased CRP level was significantly associated with shorter CSS (*p* < 0.001, log-rank test), as well as with shorter OS (*p* < 0.001, log-rank test) and loco-regional control (*p* = 0.001, log-rank test). In addition, multivariate analysis identified CRP as an independent predictor for CSS (hazard ratio (HR) 1.59, 95% confidence interval (CI) 1.08–2.35; *p* = 0.020) as well as for OS (HR 1.62, 95%CI 1.17–2.24; *p* = 0.004) and LC (HR 1.50, 95%CI 1.06–2.14; *p* = 0.023). In subgroup analysis, Kaplan Meier curves revealed that an elevated pre-treatment CRP level was a consistent prognostic factor for poor CSS (*p* = 0.003, log-rank test), OS (*p* = 0.001, log-rank test), and LC (*p* = 0.028, log-rank test) in patients treated with definitive (chemo-) radiotherapy, whereas a significant association in patients undergoing surgery and postoperative radiotherapy was not detected. The pre-treatment CRP level seems to represent a prognostic factor for CSS, OS, and LC in patients with oral and oropharyngeal cancer, particularly in those treated with definitive (chemo-) radiotherapy. Additional large-scale prospective studies are warranted to confirm and extend our findings.

## 1. Introduction

Oral and oropharyngeal squamous cell carcinoma (OOSCC) represents a major cause of morbidity and mortality worldwide. At the time of diagnosis, approximately two-thirds of patients present with advanced-stage disease with either loco-regional spread to the lymph nodes or distant metastasis [1]. Within two years after treatment, up to 50% of patients experience loco-regional recurrence with limited options for salvage surgery or re-irradiation treatment [2].

The prognosis of OOSCC depends on biological cancer characteristics as well as on patient and treatment characteristics. During recent years, a large number of translational research studies have revealed an association of various molecular biomarkers with clinical outcome in OOSCC. Molecular predictors of prognosis (e.g., DNA methylation markers or miRNA expression) may provide additional prognostic and/or predictive information, but high costs, lack of standardization and regional availability limit their application in routine clinical practice [3,4].

A hallmark of many cancers, including head and neck squamous cell carcinoma (HNSCC), is the presence of a tumor promoting phenotype of chronic, low-grade cancer-related inflammation [1,2]. Inflammation has been linked with cancer development and progression due to the functions of various cytokines, adhesion molecules and pro-angiogenic factors up-regulated in response to the inflammatory reaction [5,6,7]. Previous studies have demonstrated a strong impact of cancer-related inflammation in early stages of cancer, however, inflammatory mediators have also been associated with tumor progression and metastasization [8]. The systemic inflammatory response, which is usually measured by surrogate blood-based parameters, such as C-reactive protein, neutrophil or platelet count, has been shown to independently predict the clinical outcome of various human cancer types [9].

C-reactive protein (CRP), an acute-phase protein, is primarily synthesized by hepatocytes in response to systemic inflammation and has been demonstrated to represent a prognostic factor in various cancer entities including HNSCC [10,11,12,13,14,15,16,17,18,19,20].

For instance, Tai et al. reported that the pre-treatment CRP level was an independent prognostic factor for disease-free survival and overall survival in patients with buccal cancer [19]. In addition, Khandavilli et al. demonstrated an association between CRP and overall survival in a European cohort of 60 patients with oral cancer [20]. Katano et al. analyzed the data of 276 patients with oro-hypopharyngeal cancer treated with radiotherapy and concluded that an elevated pre-treatment CRP level was an independent predictor of both decreased CSS and OS [17]. The aim of the present study was to validate the prognostic significance of the pre-treatment CRP and to further clarify the role of the pre-treatment CRP level in a large European cohort of non-metastatic OOSCC patients treated with definitive or postoperative (chemo-) radiotherapy.

## 2. Results

### 2.1. Analysis at Baseline

A total of 503 OOSCC patients were included in the present analysis. Patient characteristics are displayed in Table 1. The median pre-treatment plasma CRP level was 5 mg/L (mean 15.08 ± 31.84 mg/L), respectively. The CRP level significantly correlated with age, alcohol abuse, pre-treatment NLR (neutrophil/lymphocyte ratio), PLR (platelet/lymphocyte ratio), tumor stage, and nodal involvement (all *p* < 0.05).

### 2.2. Analysis of Outcome

Median follow-up time was 61 months (mean 64.3 ± 2.0 months). During this period, 128 patients (25.4%) died due to OOSCC, a total of 187 patients (37.2%) died of any cause, 146 patients (29.0%) developed loco-regional recurrence. The 3- and 5- year CSS estimates were 70% and 57%, the 3- and 5- year OS probabilities were 63% and 48%, the 3- and 5- year estimates for LC were 64% and 52%, respectively.

### 2.3. Predictors of Outcome

In univariate analysis, the pre-treatment CRP level was significantly associated with CSS (HR 1.009, 95% CI 1.005–1.013; *p* < 0.001), OS (HR 1.008, 95% CI 1.003–1.013; *p* = 0.002), and LC (HR 1.007, 95% CI 1.003–1.012; *p* = 0.002). Furthermore, univariate analysis identified BMI (*p* = 0.003), smoking status (*p* = 0.028), alcohol abuse (*p* = 0.001), primary tumor site (*p* = 0.001), tumor stage (*p* < 0.001), surgery (*p* < 0.001), and the pre-treatment PLR (*p* = 0.038) as significant prognostic factors for CSS. In addition, a significant association of the BMI (*p* < 0.001), smoking status (*p* = 0.003), alcohol abuse (*p* < 0.001), tumor stage (*p* < 0.001), and surgery (*p* < 0.001) with OS has been detected. Furthermore, univariate analysis identified BMI (*p* = 0.003), smoking status (*p* = 0.016), alcohol abuse (*p* = 0.003), primary tumor site (*p* < 0.001), tumor stage (*p* < 0.001), and surgery (*p* < 0.001) as significant prognostic factors for LC. Results of univariate analyses are given in Table 2.

For further analyses, patients were grouped into those with normal CRP levels (<5.0 mg/L) and those with elevated CRP levels (≥5.0 mg/L). Overall, there were 256 patients (50.9%) with a normal (<5.0 mg/L) CRP level and 247 patients (49.1%) with an elevated (≥5.0 mg/L) CRP level.

**Figure 1:**
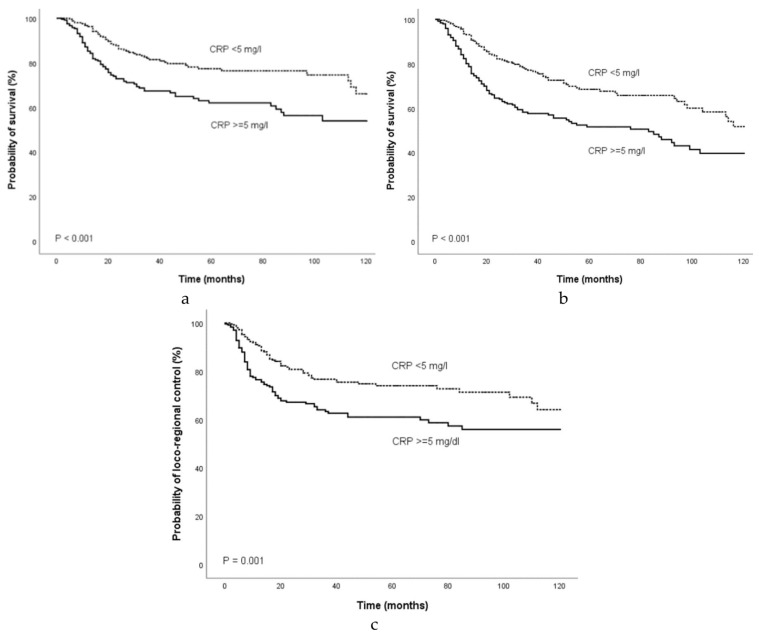
Kaplan-Meier curves for (**a**) cancer-specific survival, (**b**), overall survival, and (**c**) loco-regional control categorized by the pre-treatment CRP level. Abbreviation: CRP, C- reactive protein.

Univariate Cox regression analysis demonstrated that a CRP level ≥5.0 mg/L was a significant prognostic factor for decreased CSS (HR 1.95, 95%CI 1.37–2.78; *p* < 0.001), OS (HR 1.85, 95%CI 1.38–2.47; *p* < 0.001) and LC (HR 1.73, 95%CI 1.25–2.40; *p* = 0.001). Figure 1 shows the Kaplan-Meier curves for CSS, OS, and LC and reveal that an elevated CRP level is a consistent factor for poor CSS (*p* < 0.001, log-rank test), OS (*p* < 0.001, log-rank test), and LC (*p* = 0.001, log-rank test) in OOSCC patients.

After adjustment for factors significantly associated with prognosis in univariate analysis, the association between an elevated CRP level and CSS remained statistically significant in multivariate analysis (HR 1.59, 95%CI 1.08-2.35; *p* = 0.020, Table 3). Multivariate analyses also showed that an elevated CRP level (≥5.0 mg/L) was an independent prognostic factor for OS (HR 1.62, 95%CI 1.17–2.24; *p* = 0.004) and LC (HR 1.50, 95%CI 1.06–2.14; *p* = 0.023, Table 3). The evaluation of the CRP level as continuous variable also revealed a significant association between the CRP level and CSS (HR 1.01, 95%CI 1.003–1.02; *p* = 0.003), OS (HR 1.01, 95%CI 1.01–1.01; *p* < 0.001) and LC (HR 1.01, 95%CI 1.003–1.01; *p* = 0.002).

### 2.4. Prognostic Role of the CRP Depending on Initial Treatment

Furthermore, the prognostic role of pre-treatment CRP level was evaluated in patients treated with definitive (chemo-) radiotherapy (n = 242) separately from those treated with surgery plus postoperative (chemo-) radiotherapy (n = 261).

**Figure 2:**
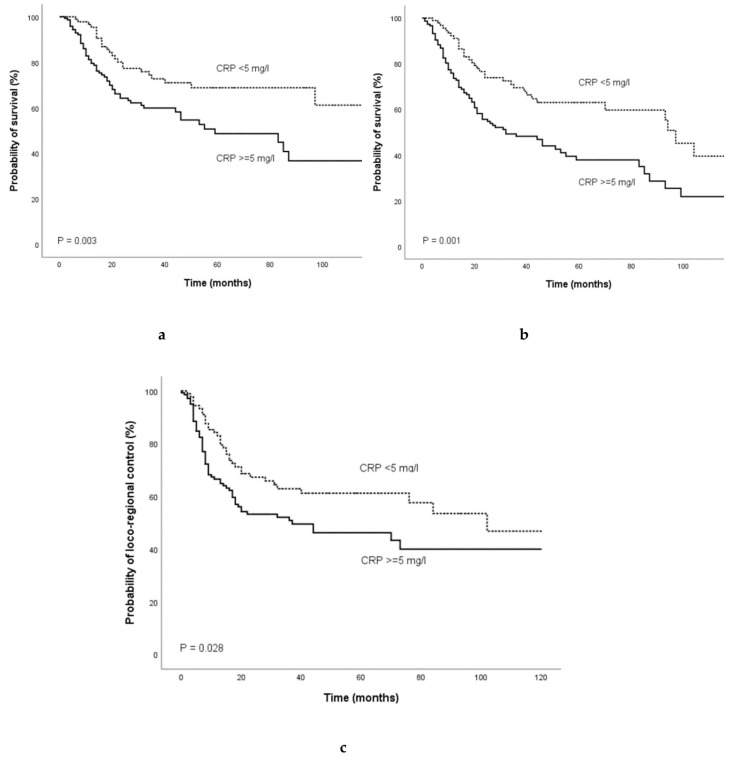
Kaplan-Meier curves for (**a**) cancer-specific survival, (**b**), overall survival, and (**c**) loco-regional control in patients treated with definitive (chemo-) radiotherapy categorized by the pre-treatment CRP level. Abbreviation: CRP, C- reactive protein.

In patients treated with definitve (chemo-) radiotherapy, Kaplan Meier curves revealed that an elevated pre-treatment CRP was a consistent prognostic factor for poor CSS (*p* = 0.003, log-rank test), OS (*p* = 0.001, log-rank test), and LC (*p* = 0.028, log-rank test) as shown in Figure 2.

Similarly, univariate analysis showed that the elevated CRP level was a significant prognostic factor for CSS (HR 2.01, 95%CI 1.25–3.25; *p* = 0.004), OS (HR 2.00, 95%CI 1.33–2.99; *p* = 0.001), and LC (HR 1.57, 95%CI 1.04–2.37; *p* = 0.031). Furthermore, a significant association of BMI (*p* = 0.010), smoking status (*p* = 0.010), alcohol abuse (*p* = 0.002), tumor site (*p* = 0.015) with CSS was shown. In addition, a significant relationship between BMI (*p* = 0.005), smoking status (*p* = 0.032), alcohol abuse (*p* < 0.001), as well as concomitant chemotherapy (*p* = 0.030) and OS was observed. Univariate analysis also revealed that BMI (*p* = 0.013) smoking status (*p* = 0.002), alcohol abuse (*p* < 0.001), tumor site (*p* = 0.001), and concomitant therapy (*p* = 0.006) were significantly associated with LC. None of the remaining parameters were significantly associated CSS, OS or LC. In multivariate analysis, an elevated CRP ≥ 5.0 mg/L level remained a significant predictor of CSS (HR 1.77, 95%CI 1.09–2.89; *p* = 0.022), OS (HR 1.75, 95%CI 1.15–2.67; *p* = 0.009), LC (HR 1.55, 95%CI 1.01–2.39; *p* = 0.046). Multivariate analyses of parameters associated with prognosis in patients treated with definitive (chemo-) radiotherapy are displayed in Table 4.

In patients treated with surgery and postoperative (chemo-) radiotherapy, the pre-treatment CRP level was not associated with CSS (HR 1.29, 95%CI 0.72–2.30; *p* = 0.394), OS (HR 1.24, 95%CI 0.78–1.97; *p* = 0.361), or LR (HR 1.15, 95%CI 0.63–2.08; *p* = 0.654).

Univariate analysis showed a significant association of tumor site (*p* < 0.001) with with CSS, in addition, a significant relationship between BMI (*p* = 0.008), smoking status (*p* = 0.032), alcohol abuse (*p* = 0.008), as well as tumor site (*p* = 0.020) and OS was observed. Univariate analysis also revealed that tumor site (*p* < 0.001) and concomitant therapy (*p* = 0.014) were significantly associated with LC.

## 3. Discussion

In the present study, we analyzed the prognostic significance of the pretreatment CRP level in patients with OOSCC treated with definitive or postoperative (chemo-) radiotherapy and detected a significant association between an elevated CRP level and poor CSS, OS, and LC.

Tumor progression and metastasization is strongly influenced by the tumor microenvironment that plays an essential role for tumor cell survival, proliferation, and migration, additionally, it contributes to the initiation of a systemic inflammatory response [6,21]. Different cytokines produced by tumor cells lead to an accumulation of leukocytes that synthesize cytokines and cytotoxic mediators including interleukin (IL)-1, IL-6, tumor necrosis factor-α (TNF-α), transforming growth factor-β (TGF-β), and interferons [22,23] that have been linked with the promotion of cancer cell proliferation, growth, and migration as well as with the stimulation of CRP production. Thus, an increase in CRP may represent elevated levels of pro-inflammatory cytokines that contribute to a microenvironment supporting tumor angiogenesis, proliferation, growth and metastases [24,25,26]. Elevated CRP concentrations have also been associated with an increase in serum levels of vascular endothelial growth factor (VEGF), which is upregulated in response to tumor hypoxia, a condition that upregulates many processes stimulating invasion and distant tumor spread and contributes to establishing an immunosuppressive tumor microenvironment [27,28]. Additionally, CRP may promote tumor growth protecting tumor cells from drug-induced apoptosis [29].

Previous studies have suggested that CRP elevation might not only be derived from an increased production in hepatocytes as a response to cancer-related systemic inflammation, but could also represent a result of CRP production in malignant cells themselves [30]. This hypothesis is supported by previous findings demonstrating an association between CRP levels and tumor size [31]. Similarly, the results of our study revealed a significant relationship between an increase in plasma CRP and advanced tumor stage.

An association between elevated CRP levels and poor prognosis has been detected in different cancer entities including HNSCC. For instance, Peter and colleagues observed a significant relationship of an elevated CRP level with advanced tumor stage, nodal status and overall survival in a cohort of 261 HNSCC patients [32]. Grimm et al. reported that CRP levels were associated with disease-free survival as well as advanced tumor stage in patients with oral cancer [33]. In addition, a Taiwanese group published various studies on the prognostic role of CRP in oral cancer and could consistently demonstrate an association between elevated CRP levels and survival [34,35,36]. More recently, pre-therapeutic indices of systemic inflammation based on CRP and albumin have been found to provide prognostic information in nasopharyngeal and laryngeal cancer patients [37,38].

Because of their previously proposed prognostic role, the pre-treatment NLR and PLR have been analyzed in order to provide a comprehensive consideration of several potential confounders. The evaluation of the NLR and PLR was a secondary objective and as there are no standardized cut-off values, we decided to analyze the NLR and PLR as continuous variables. In the present study, we did not observe a significant association between an elevated NLR or PLR and OOSCC prognosis. In contrast, many previous studies have shown that both NLR and PLR are significant predictors of outcome. Their numerator, the neutrophil, and platelet counts are considered as adverse outcome predictors, whereas the lymphocyte count in the denominator is considered to represent a favorable outcome predictor and higher circulating lymphocyte counts have been associated with improved survival. However, lymphocyte exhaustion and dysfunction have well been described in HNSCC, therefore, lymphocyte counts may serve as an unreliable prognostic parameter [39]. Furthermore, most of the included studies on the prognostic role of the NLR and PLR were performed in Asians, thus, the results about non-Asians should be interpreted cautiously and need to be validated further. Additionally, despite the lack of standardized cut-off values, most of the studies were performed using cut-off levels ranging from 1.62 to 5 for the NLR and 81.62 to 300 for the PLR [40].

Few studies have investigated the prognostic impact of the pre-treatment CRP level in HNSCC patients treated with radiotherapy. For instance, Katano et al. analyzed the prognostic role in patients with oro-hypopharyngeal cancers undergoing definitive radiotherapy and reported an association between an elevated CRP level and decreased CSS, OS as well as LC [17]. In contrast, Kruse et al., who evaluated the prognostic significance of the pre-treatment CRP in a cohort of 278 patients treated with primary surgery, did not detect a significant relationship between an elevated CRP level (>5 mg/L) and disease-free or overall survival [41]. In our study, we observed a significant association between an elevated CRP level and poor prognosis in patients treated with definitive (chemo-) radiotherapy, whereas a significant relationship was not observed after primary surgery and postoperative (chemo-) radiation. Previously, Selzer et al. evaluated the prognostic role of inflammatory parameters in patients treated with definitive or postoperative radiotherapy [42]. Similar to our findings, the authors detected a significant prognostic role of inflammatory factors in patients treated with definitive radiotherapy, but could not find a significant role in those who had undergone surgery and postoperative radiotherapy. The reason for this finding is speculative but might be explained by the substantial role of inflammatory and immunological mechanisms for the mediation of radiation- induced effects.

Remarkable systemic immunological consequences have been observed in patients receiving radiotherapy to control tumors [43]. Radiation alters the balance between immune-activating and suppressive signals in the tumor microenvironment, in addition, tumor-targeted radiation has been found to promote antitumor T cell responses that mediate local tumor rejection and also elicit immune-mediated systemic tumor regression [44]. Galluzzi and colleagues described molecular pathways that are activated during cell stress in response to radiation and contribute to a type of cell death that is immunogenic, enabling signaling to the immune system and may result in the acquisition of a tumor-specific immunity, so that both the primary tumor and sites of metastases are attacked [45]. However, effects outside the field of local radiotherapy have primarily been observed after definitive irradiation of tumors. Patients treated with definitive radiotherapy have been found to acquire tumor-specific immune responses as demonstrated by new antibody formation, this effect has been detected after radical surgical treatment to a lesser extent [46]. The presence of cancer-related systemic inflammation reflected by an increased CRP level might stronger interact with systemic responses caused by primary tumor-directed radiation and therefore play a more prominent role for prognosis in patients treated with definitive radiotherapy compared to those having undergone radical surgery before radiotherapy.

Anti-inflammatory therapy has been proposed to be an effective strategy for cancer treatment. In individuals taking aspirin and non-steroidal anti-inflammatory drugs (NSAIDs), a lower incidence of colon cancer has been detected in previous observational studies as well as randomized trials [47,48]. Inflammatory factors may promote the initiation and progression of various types of cancer, thus, NSAIDs have been suggested to reduce cancer incidence as well as to prevent cancer progression by interacting with various inflammatory pathways [49]. Thus, treatment with NSAIDs could be beneficial in enhancing treatment effects in cancer patients, particularly in those treated with definitive (chemo-) radiotherapy [50].

Our findings support the hypothesis that the pre-treatment CRP level might be associated with prognosis in OOSCC patients. However, some limitations of the present investigation have to be taken into consideration. In view of the retrospective design of our investigation, we cannot completely exclude a selection bias and the possibility of an unequal distribution of unidentified clinico-pathological parameters that may have caused a bias of the observed results. Additionally, CRP represents a non-specific marker of inflammation and might have been influenced by various conditions or factors including infections, inflammatory diseases, connective tissue disorders, severe stress, and medical treatments which we could not account for in this study. Potential confounding factors known to influence plasma CRP levels may underlie the differences in overall mortality between patients with elevated and those with normal CRP levels. Another limitation may result from the long time period during which the included patients have been treated. Several improvements in diagnostic and therapeutic procedures as well as advances in CRP determination have been introduced from 2000 to 2017.

To the best of our knowledge, the present study is currently the largest one investigating the prognostic value of the plasma CRP levels in HNSCC patients. Furthermore, CSS that directly reflects OOSCC prognosis has been defined as primary endpoint and has been found to be significantly influenced by pre-treatment CRP levels, particularly in patients treated with definitive (chemo-) radiotherapy.

Nevertheless, due to the retrospective nature of the present study, our results have to be regarded as preliminary. Validation of our data in additional prospective large-scale studies is imperative before firm conclusions about the role of CRP for prognosis in OOSCC patients can be drawn. If confirmed by additional studies, determination of plasma CRP levels may contribute to the identification of patients who might be candidates for additional, more aggressive treatment approaches also including anti- inflammatory drugs or more stringent follow-up schedules.

## 4. Materials and Methods

Data from 503 consecutive patients with histologically confirmed OOSCC treated at the Department of Therapeutic Radiology and Oncology Graz from 2000 to 2017 were analyzed retrospectively. Patients enrolled in the present study fulfilled the following eligibility criteria: (a) histologically confirmed primary squamous cell carcinoma; (b) no evidence of distant metastasis; (c) treatment for curative intent; and (d) no evidence of other malignancies. At the time of diagnosis, each patient was routinely referred to our interdisciplinary tumor board. A total of 261 patients were treated with postoperative (chemo-) radiotherapy. The remaining patients underwent definitive (chemo-) radiotherapy alone.

A total of 303 patients received concurrent chemotherapy, primarily consisting of a platinum-based regimen, in addition, targeted therapy such as cetuximab was also used. An induction chemotherapy consisting of docetaxel, cisplatin, 5-fluorouracil (TPF)-based was administered in 77 patients. Laboratory data, including CRP were obtained as part of routine clinical evaluation prior to the initiation of treatment and evaluated using standard clinical testing methodology. According to the current standards for CRP determination of the Clinical Institute of Medical and Chemical Laboratory Diagnostics, Medical University of Graz, a plasma CRP concentration of ≥5 mg/L was considered pathological. The neutrophil/lymphocyte ratio (NLR) was calculated as the absolute neutrophil count measured in G/L divided by the absolute lymphocyte count measured in G/L, the platelet/lymphocyte ratio (PLR) as the absolute platelet count measured in G/L divided by the absolute lymphocyte count measured in G/L, respectively. At the initiation of treatment, none of included patients showed clinical evidence of an inflammatory condition or bacterial infection. Clinical staging was performed according to the 7th edition of American Joint Committee on Cancer (AJCC) staging in oral and oropharyngeal cancer.

All patients underwent radiotherapy with 6 MV photon linear accelerators. The dose-fractionation regimen was either standard fractionation or a simultaneous integrated boost (SIB) protocol. Standard fractionation radiotherapy was delivered up to a total dose of 70.0 Gy in 35 fractions (2.0 Gy per fraction/5× per week). The prescription dose to primary lesions or positive nodes ranged from 66 to 70 Gy, prophylactic nodal areas were irradiated at doses of 50 Gy. The SIB radiation schedules consisted of 5 × 2 Gy or 5 × 2.2 Gy /week to 70 Gy or 70.4 Gy to clinically manifest sites of gross disease and 5 × 1.6 Gy or 1.69 Gy /week to 56 Gy or 54 Gy to adjacent lymphatic drainage regions at risk for subclinical metastasis.

The radiation method was either three-dimensional conformal radiotherapy or intensity modulated radiotherapy (IMRT), including volumetric modulated arc therapy (VMAT). Patients treated with postoperative radiotherapy received standard fractionation RT up to a total dose of 60–70 Gy at 2 Gy per fraction, depending on risk factors such as resection margin and tumor stage.

Follow-up examinations were performed both at the Department of Therapeutic Radiology and Oncology and at the Department of Otorhinolaryngology according to institutional guidelines. Complete physical examination was performed every 3 months (years 1–2)/ every 6 months (years 3–5), and annually thereafter, whereas imaging was performed as indicated by clinical examination.

The study complied with the Declaration of Helsinki and was performed according to the national law. The protocol has been approved by the local Ethical Committee (approval number: EK 29-273 ex 16/17 and EK 31-061 ex 18/19). As this is a retrospective non-intervening study, the Ethical Committee waived the need for written informed consent from the study participants.

CSS was defined as the primary study endpoint and calculated as the time from initiation of treatment for OOSCC to the date of patients’ OOSCC-related death. The secondary endpoints included OS defined as the time from start of treatment to the date of death of any cause and LC defined as no evidence of recurrence or progression of the primary tumor and neck lymph nodes. Normality testing was performed using the Shapiro-Wilk Test and the Kolmogorov-Smirnov Test. The non-normally distributed CRP levels prompted us to apply non-parametric tests (Mann Whitney U Test, Kruskal Wallis Test, and Spearman correlation) for the correlation analyses. According to previously published studies, an elevated CRP level ≥5 mg/L was selected as cutoff value for analysis [35,36]. The association of the CRP level and other clinico-pathological features with CSS, OS, and LC was analyzed using Kaplan-Meier curves and compared by the log-rank test. Univariate Cox proportion analysis was performed to determine the influence of an elevated CRP level and other clinico-pathological factors such as age at diagnosis, sex, BMI, smoking status, alcohol abuse, tumor site, tumor grade, tumor stage, nodal involvement, NLR, and PLR on CSS, OS and LC. Hazard ratios (HRs) estimated from the Cox proportion analysis were reported as relative risks with corresponding 95% confidence intervals (CIs). Multivariate Cox proportion analyses were applied to evaluate the impact of potential confounders on CSS, OS, and LC and included parameters significantly associated with outcome in univariate analyses. All statistical analyses were performed using the Statistical Package for Social Sciences version 25.0 (SPSS Inc., Chicago, IL, USA). A two-sided *p* < 0.05 was considered statistically significant.

## 5. Conclusions

In conclusion, the pre-treatment plasma CRP level represents a prognostic factor in OOSCC patients, particularly those treated with definitive (chemo-) radiotherapy and may support oncological therapy decisions.

## Figures and Tables

**Table 1:** Summary of patient characteristics.

Criterion	Value
Number of patients	503
Sex	
Male	378 (75.1%)
Female	125 (24.9%)
Age; median (mean ± SD)	58.00 (59.2 ± 10.7)
BMI; median (mean ± SD)	24.03 (24.52 ± 4.39)
Smoking status	
Former * or never	183 (36.4%)
Current	313 (62.2%)
Alcohol abuse	
Former * or never	292 (58.1%)
Current	197 (39.2%)
Tumor site	
Oral cavity	195 (38.8%)
Oropharynx	308(61.2%)
Tumor grade	
G 1/2	249 (49.5%)
G 3/4	249 (49.5%)
Tumor stage	
T 1/2	201 (40.0%)
T 3/4	292 (58.1%)
Nodal involvement	
Yes	401 (79.7%)
No	96 (19.1%)
Surgery	
Yes	261 (51.9%)
No	242 (48.1%)
Induction chemotherapy	
Yes	77 (15.3%)
No	426 (84.7%)
Concomitant chemotherapy	
Yes	303 (60.2%)
No	199 (39.6%)
NLR, median (mean ± SD)	3.15 (3.79 ± 2.66)
PLR, median (mean ± SD)	155.8 (178.1 ± 99.42)
CRP, median (mean ± SD)	5.0 (15.08 ± 31.84)

* Former smoking or alcohol abuse was defined as tobacco or alcohol abuse before or until the start of treatment. Abbreviations: BMI, body mass index; NLR, neutrophil/lymphocyte ratio; PLR, platelet/lymphocyte ratio; CRP, C-reactive protein; SD, standard deviation.

**Table 2:** Univariate analysis of clinical-pathological parameters for the prediction of cancer-specific survival, overall survival and loco-regional control.

Criterion	Cancer–Specific Survival		Overall Survival		Loco-Regional Control	
	HR (95% CI) *	*p*-Value	HR (95% CI)	*p*-Value	HR (95% CI)	*p*-Value
Sex						
Male	1		1		1	
Female	1.15 (0.78–1.70)	0.469	0.93 (0.67–1.30)	0.681	1.09 (0.76–1.57)	0.636
Age (continuous)	1.01 (0.99–1.02)	0.412	1.01 (0.996–1.02)	0.191	1.01 (0.99–1.02)	0.508
BMI (continuous)	0.93 (0.89–0.98)	0.003	0.92 (0.88–0.96)	<0.001	0.94 (0.90–0.98)	0.003
Smoking status						
Former/never	1		1		1	
Current	1.54 (1.05–2.25)	0.028	1.62 (1.18–2.24)	0.003	1.55 (1.08–2.21)	0.016
Alcohol abuse						
Former/never	1		1		1	
Current	1.83 (1.29–2.59)	0.001	2.04 (1.52–2.73)	<0.001	1.65 (1.19–2.29)	0.003
Tumor site						
Oropharynx	1		1		1	
Oral cavity	1.82 (1.29–2.58)	0.001	1.29 (0.96–1.72)	0.092	1.90 (1.37–2.63)	<0.001
Tumor grade						
G 1/2	1		1		1	
G 3/4	1.04 (0.74–1.48)	0.807	0.98 (0.73–1.31)	0.889	0.89 (0.65–1.24)	0.502
Tumor stage						
T 1/2	1		1		1	
T 3/4	2.24 (1.52–3.31)	<0.001	1.96 (1.43–2.68)	<0.001	2.80 (1.91–4.12)	<0.001
Nodal involvement						
No	1		1		1	
Yes	1.07 (0.68–1.70)	0.774	1.02 (0.70–1.48)	0.928	0.95 (0.63–1.43)	0.787
Surgery						
No	1		1		1	
Yes	0.39 (0.27–0.56)	<0.001	0.43 (0.32–0.58)	<0.001	0.29 (0.21–0.42)	<0.001
Induction chemotherapy						
No	1		1		1	
Yes	1.52 (1.001–2.29)	0.049	1.20 (0.83–1.73)	0.341	1.61 (1.09–2.38)	0.016
Concomitant chemotherapy						
No						
Yes	0.98 (0.68–1.40)	0.898	1.05 (0.78–1.42)	0.738	0.94 (0.68–1.31)	0.721
NLR (continuous)	1.035 (0.975–1.098)	0.257	1.047 (0.998–1.097)	0.059	1.026 (0.969–1.086)	0.38
PLR (continuous)	1.002 (1.000–1.003)	0.038	1.001 (1.000–1.002)	0.149	1.001 (1.000–1.003)	0.186
CRP (continuous)	1.009 (1.005–1.013)	<0.001	1.008 (1.003–1.013)	0.002	1.007 (1.003–1.012)	0.002
CRP						
<5	1		1		1	
≥5	1.95 (1.37–2.78)	<0.001	1.85 (1.38–2.47)	<0.001	1.73 (1.25–2.40)	0.001

* Abbreviations: CI = confidence interval; HR = hazard ratio; BMI, body mass index; NLR, neutrophil/lymphocyte ratio; PLR, platelet/lymphocyte ratio; CRP, C-reactive protein.

**Table 3:** Multivariate analysis of clinical-pathological parameters for the prediction of cancer-specific survival, overall survival and loco-regional control.

Criterion *	Cancer-Specific Survival		Overall Survival		Loco-Regional Control	
	HR (95% CI)	*p*-Value	HR (95% CI)	*p*-Value	HR (95% CI)	*p*-Value
BMI (continuous)	0.96 (0.92–1.01)	0.134	0.93 (0.90–0.97)	0.001	0.97 (0.93–1.01)	0.181
Smoking status						
Former/never	1		1		1	
Current	1.08 (0.67–1.74)	0.748	1.07 (0.72–1.59)	0.723	1.15 (0.75–1.78)	0.526
Alcohol abuse						
Former/never	1		1		1	
Current	1.55 (1.03–2.34)	0.037	1.55 (1.10–2.20)	0.012	1.52 (1.04–2.21)	0.03
Tumor site						
Oropharynx	1				1	
Oral cavity	1.99 (1.34-2.94)	0.001	-	-	2.30 (1.59–3.32)	<0.001
Tumor stage						
T 1/2	1		1		1	
T 3/4	1.16 (0.70–1.91)	0.565	1.02 (0.68–1.54)	0.919	1.67 (1.04–2.68)	0.035
Surgery						
No	1		1		1	
Yes	0.39 (0.23–0.64)	<0.001	0.460 (0.31–0.69)	<0.001	0.34 (0.22–0.55)	<0.001
Induction chemotherapy						
No	1				1	
Yes	1.08 (0.67–1.75)	0.76	-	-	1.06 (0.68–1.65)	0.804
PLR (continuous)	1.001 (0.999-1.003)	0.239	-	-	-	-
CRP						
<5	1		1		1	
≥5	1.60 (1.08–2.37)	0.019	1.62 (1.17–2.24)	0.004	1.51 (1.60–2.15)	0.023

* Multivariate Cox proportion analysis included parameters significantly associated with outcome in univariate analyses. Abbreviations: CI = confidence interval; HR = hazard ratio; BMI, body mass index; NLR, neutrophil/lymphocyte ratio; PLR, platelet/lymphocyte ratio; CRP, C-reactive protein.

**Table 4:** Multivariate analysis of clinical-pathological parameters for the prediction of cancer-specific survival, overall survival and loco-regional control in patients treated with definitive (chemo-) radiotherapy.

Criterion *	Cancer-Specific Survival		Overall Survival		Loco-Regional Control	
	HR (95% CI)	*p*-Value	HR (95% CI)	*P*-Value	HR (95% CI)	*p*-Value
BMI (continuous)	0.96 (0.90–1.01)	0.135	0.94 (0.90–0.99)	0.015	0.97 (0.92–1.02)	0.233
Smoking status						
Former/never	1		1		1	
Current	1.18 (0.66–2.09)	0.574	0.94 (0.59–1.51)	0.806	1.34 (0.80–2.23)	0.269
Alcohol abuse						
Former/never	1		1		1	
Current	1.80 (1.10–2.92)	0.019	1.79 (1.17–2.74)	0.007	1.80 (1.16–2.80)	0.009
Primary site						
Oropharynx	1				1	
Oral cavity	1.65 (1.02–2.67)	0.041	-	-	1.73 (1.1–2.73)	0.018
Concomitant chemotherapy						
No			1		1	
Yes	-	-	0.55 (0.35–0.87)	0.01	0.59 (0.36–0.97)	0.037
CRP						
<5	1		1		1	
≥5	1.77 (1.09–2.89)	0.022	1.75 (1.15–2.67)	0.009	1.55 (1.01–2.39)	0.046

* Multivariate Cox proportion analysis included parameters significantly associated with outcome in univariate analyses. Abbreviations: CI = confidence interval; HR = hazard ratio; BMI, body mass index; NLR, neutrophil/lymphocyte ratio; PLR, platelet/lymphocyte ratio; CRP, C-reactive protein.
